# Distinctive Prognostic Value and Cellular Functions of Osteopontin Splice Variants in Human Gastric Cancer

**DOI:** 10.3390/cells10071820

**Published:** 2021-07-19

**Authors:** Chengcheng Hao, Yuxin Cui, Jane Lane, Shuqin Jia, Jiafu Ji, Wen G. Jiang

**Affiliations:** 1 Department of Oncology, Beijing Shijitan Hospital, Capital Medical University, Beijing 100038, China; Haochengcheng@bjsjth.cn; 2 Cardiff China Medical Research Collaborative, Cardiff University School of Medicine, Heath Park, Cardiff CF14 4XN, UK; CuiY7@cardiff.ac.uk (Y.C.); Lanej1@cardiff.ac.uk (J.L.); 3 Key Laboratory of Carcinogenesis and Translational Research (Ministry of Education), Peking University Cancer Hospital and Institute, No. 52, Beijing 100142, China; shuqin_jia@hsc.pku.edu.cn (S.J.); jiafuji_pkuos@sina.com (J.J.)

**Keywords:** osteopontin, OPN splice variants, gastric cancer, prognosis, biological function, chemoresistance

## Abstract

Background: Osteopontin (OPN) splice variants are identified as predictors of tumour progression and therapeutic resistance in certain types of solid tumours. However, their roles in gastric cancer (GC) remain poorly characterized. The current study sought to assess the prognostic value of the three OPN splice variants (namely OPN-a, OPN-b, and OPN-c) in gastric cancer and their potential functions within gastric cancer cells. Methods: RNA extraction and reverse transcription were performed using our clinical cohort of gastric carcinomas and matched normal tissues (n = 324 matched pairs). Transcript levels were determined using real-time quantitative PCR. Three OPN splice variants overexpressed cell lines were created from the gastric cancer cell line HGC-27. Subsequently, biological functions, including cell growth, adhesion, migration, and invasion, were studied. The potential effects of OPN isoforms on cisplatin and 5-Fu were evaluated by detecting cellular reactive oxygen species (ROS) levels in the HGC-27-derived cell lines. Results: Compared with normal tissues, the expression levels of three splice variants were all elevated in gastric cancer tissues in an order of OPN-a > OPN-b > OPN-c. The OPN-a level significantly increased with increasing TNM staging and worse clinical outcome. There appeared to be a downregulation for OPN-c in increasing lymph node status (*p* < 0.05), increasing TNM staging, and poor differentiation. High levels of OPN-a and OPN-b were correlated with short overall survival and disease-free survival of gastric cancer patients. However, the low expression of OPN-c was significantly associated with a poor prognosis. Functional analyses further showed that ectopic expression of OPN-c suppressed in vitro proliferation, adhesiveness, migration, and invasion properties of HGC-27 cells, while the opposite role was seen for OPN-a. Cellular ROS detection indicated that OPN-a and OPN-c significantly promoted ROS production after treatment with 5-Fu comparing to OPN-vector, while only OPN-a markedly induced ROS production after treatment with cisplatin. Conclusion: Our results suggest that OPN splice variants have distinguished potential to predict the prognosis of gastric cancer. Three OPN variants exert distinctive functions in gastric cancer cells. Focusing on specific OPN isoforms could be a novel direction for developing diagnostic and therapeutic approaches in gastric cancer.

## 1. Introduction

Gastric cancer (GC) is one of the most commonly diagnosed cancers and the leading cause of death worldwide, especially in Far East Asia [1]. According to the statistics provided by the World Health Organization, approximately one million new cases of gastric cancer and more than 8% of deaths worldwide from cancer occurred in 2018. Despite multiple treatment methods including surgery, chemotherapy, radiotherapy, and targeted therapy which have been applied over the past few decades [2], the prognosis of patients with gastric cancer remains poor with survival rates around 30% worldwide [3]. The conventional predictive factors, such as TNM stage, age, sex, and histological type are valuable, but molecular prognostic factors have been an area of intense research. Therefore, it is necessary to exploit novel biomarkers that could accurately predict the outcome to guide clinical management and improve the prognosis of patients with gastric cancer.

As a phosphorylated glycoprotein, Osteopontin (OPN/SPP1) is secreted by various tissues and cells, and has been implicated in physiological as well as pathological processes. Over the last few years, accumulating evidence has shown that aberrant OPN expression is closely associated with tumourigenesis and metastasis in several tumours, including gastric cancer [4,5]. Multiple malignant gastric cancer cell lines overexpress the metastatic related gene osteopontin, and the transfection of this gene into benign tumorigenic gastric epithelial cell lines conveys invasive behaviour. Although several studies have explored the association between OPN expression and the clinical outcome and prognosis of gastric cancer patients, the results regarding the ability of OPN to predict disease progression in patients with gastric cancer emerged controversial [6].

The biological functions of tumour-associated gene products are extensively regulated on posttranscriptional levels. Alternative splice-generation of multiple mRNA products from a single gene is a critical mechanism for generating proteomic diversity. Consistently, the OPN precursor-mRNA (pre-mRNA) is subject to alternative splicing, which generates three splice variants, OPN-a (consists of all exons), OPN-b (lacks exon 5), and OPN-c (lacks exon 4). Recent studies have shown that OPN splice variants are differentially expressed and may have functional heterogeneity in a tumor-specific manner [7,8,9]. For example, OPN-b expression dominates in gliomas, but is hardly detected in breast cancer tissues. Overexpression of OPN-c promotes tumour metastasis in ovarian cancer, but prevents both cell migration and invasion in hepatocellular carcinoma. Based on these findings, we postulate that a specific OPN isoform might become more valuable as a cancer target than OPN itself, since its diverse roles are dependent on the cancer type. However, up to date, few studies have focused on three OPN splice variants in gastric cancer.

In the present study, we aimed to investigate the clinical significance of the OPN splice variants by taking advantage of our gastric cancer cohort. The roles of the three OPN splice variants in gastric cancer cells and in cells’ response to chemotherapeutic agents were also assessed. 

## 2. Materials and Methods

### 2.1. Patient Specimens

A total of 324 patients (male 231, female 93; mean age 59.8 years; range 23–87 years) with gastric cancer, who were diagnosed and surgically treated at the Peking University Cancer Hospital between 2004 and 2007, were enrolled in this study. The study was approved by the local ethics committee (Ethics Number: 2006021) and consent was obtained from patients. Some of the patients had received chemotherapy or radiation therapy preoperatively. The following histopathological information was collected: depth of tumour invasion, histological grade, status of lymph node metastasis, presence or absence of liver metastases and vascular invasion. The staging of gastric cancer was classified according to the 1997 tumour node metastasis (TNM) classification recommended by the International Union Against Carcinoma. All patients were followed up until June 2012.

### 2.2. Cell Lines

Human gastric cancer cell lines, AGS and HGC-27, were acquired from the European Collection of Animal Cell Culture (Salisbury, England, UK). Cells were maintained in DMEM-F12 medium supplemented with 10% fetal bovine serum and antibiotics.

### 2.3. Plasmid Construction and Cell Transfection

OPNs’ cDNAs were respectively cloned into the GV141 vector by Genechem Co., Ltd. (Shanghai, China) to establish the OPN isoform-overexpressing plasmids. Cells were transfected with corresponding plasmids by using the Lipo3000 reagent (L3000015, Invitrogen, Waltham, MA, USA) according to the manufacturer’s instruction. OPN expression was verified by RT-PCR after cells were transfected for 24 h.

### 2.4. Reverse Transcription-Polymerase Chain Reaction (RT-PCR)

RNA extraction, reverse transcription (RT), and polymerase chain reaction were performed as described previously [10]. The reactions were performed with denaturing for 15 s at 94 °C, annealing for 30 s at 58 °C, and elongation for 30 s at 72 °C for a total of 30 cycles; the final extension was done at 72 °C for 5 min. PCR products were analyzed by Tris-acetate EDTA agarose (1% *w*/*v*) gel electrophoresis.

### 2.5. RNA Extraction and Real-Time Quantitative Polymerase Chain Reaction (RT-QPCR)

RNA extraction, reverse transcription (RT) and RT-QPCR were performed as described previously [10]. Z- sequence on RT-QPCR primers was 5′-ACTGAACCTGACCGTACA-3′, which is complementary to the universal Z probe used for the RT-QPCR (TCS Biological Ltd., Oxford, UK). Real-time RT-QPCR conditions were 95 °C for 15 min, followed by 60 cycles at 95 °C for 20 s, 55 °C for 30 s, and 72 °C for 20 s. The sequences of the genes are listed in Table 1.

### 2.6. In Vitro Cell Growth Assay

A standard procedure was used as previously described [11]. Gastric cells were plated into a 96-well plate (3000 cells per well). Cells were fixed in 4% formalin after 1, 2, 3, and 4 days. The cells were then stained with 0.5% (*w*/*v*) crystal violet for half an hour. Following washing, stained crystal violet was extracted with 10% (*v*/*v*) acetic acid. Absorbance was determined at a wavelength of 540 nm using a spectrophotometer (Elx800; Bio-Tek, Bedfordshire, UK).

### 2.7. In Vitro Invasion Assay

This was carried out as previously reported and modified in our laboratory [12]. Transwell inserts with an 8-µm pore size were coated with 50 mg Matrigel and air-dried. The Matrigel was rehydrated before use. A total of 25,000 cells were seeded to each well and after 48 h, cells that had migrated through the matrix and pores were fixed with 4% formalin, stained in crystal violet, and counted.

### 2.8. In Vitro Cell–Matrix Adhesion Assay

A total of 40,000 cells were added to each well of a 96-well tissue-culture plates previously prepared by coating with Matrigel (5 mg per well). The cells were incubated at 37 °C in 5% CO_2_ for 40 min and the medium was then discarded. Nonadherent cells were washed off using PBS buffer. The remaining cells were then fixed in 4% formaldehyde for 5 min. After further washing, cells were stained with crystal violet, and the number of adherent cells was then counted.

### 2.9. Electrical Cell Substrate Impedance Sensing (ECIS)-Based Cell Migration Assay

This method has been widely implemented by our laboratories as previously reported [13]. Briefly, 96-well W96E1 microarrays were used on the ECIS^®^Zθ (theta) instrument (Applied Biophysics Ltd., Troy, New Jersey, USA). Cells were added to the wells of the array. Then, confluent lung cancer monolayers in the arrays were electrically wounded (2000 mA for 20 s each), after which the migration of the cells was immediately tracked, again over a range of frequencies (1000 Hz–64,000 Hz). All the experiments were conducted in triplicate.

### 2.10. Measurement of ROS Production

Cellular ROS generation was measured with a Cellular Reactive Oxygen Species Detection Assay Kit (Red Fluorescence) (Abcam, UK). HGC-27 (2.5 × 10^4^ cells). Cells which overexpressed OPN splice variants were seeded into 96-well plates and treated with the drugs (cisplatin and 5-Fu, respectively), incubated for 24 h in the dark and ROS Red Dye Working Solution was then added. After incubating at 37 °C for 30 min, the fluorescence intensity increase was monitored at an excitation wavelength of 650 nm and an emission wavelength of 675 nm with the bottom read mode.

### 2.11. Protein Prediction

The protein sequences were obtained from https://www.uniprot.org/uniprot (accessed 28 April 2021). The identifiers of the three OPN splice variants were P10451-1 (OPN-a), P10451-5 (OPN-b), and P10451-3 (OPN-c), respectively. The prediction of the protein features was conducted using https://predictprotein.org (accessed 28 April 2021).

### 2.12. Statistical Analysis

Data of gene expression from our gastric cancer cohort were analysed using Minitab (version 14. Minitab Ltd., Coventry, UK). Mann–Whitney U tests or Kruskal–Wallis tests were used to compare gene expression between patient groups. The association of the OPN splice variants with the patient survival was evaluated using Kaplan–Meier survival analysis. The prognostic values of the factors were undertaken by univariate and multivariate analysis of variance. The *in-vitro* cellular function data were analysed by R programming (version 4.0.5. https://www.r-project.org, accessed on 28 April 2021). Following normality test, Student’s t-test was used for the comparison of two groups while ANOVA was used for multiple groups. A *p* < 0.05 was regarded as statistically significant unless otherwise indicated. The statistical asterisks are: * *p* < 0.05, ** *p* < 0.01, and *** *p* < 0.001. 

## 3. Results

### 3.1. The Prognostic Value of OPN Splice Variants in Gastric Cancer Patients

**Figure 1:**
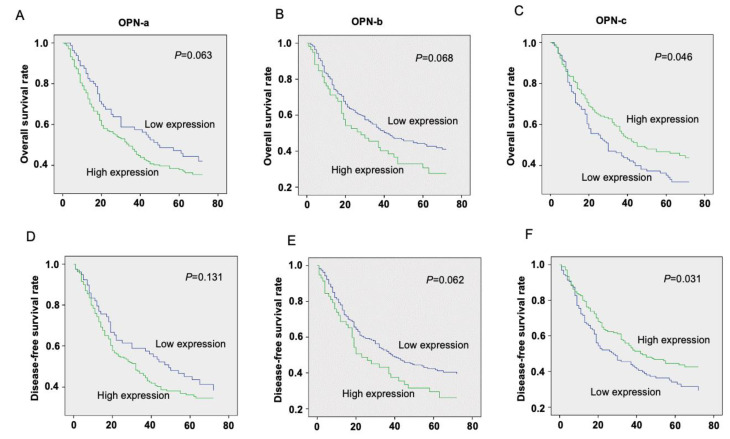
Prognosis values of OPN splice variants’ expression in gastric cancer patients. The OS and DFS of the gastric cancer patients were positively associated with the overexpression of OPN-a (**A**,**D**) and OPN-b (**B**,**E**), individually. While decreased OPN-c expression was significantly associated with adverse pathology in gastric cancer (**C**,**F**).

The Kaplan–Meier survival curve indicated that gastric cancer patients with high OPN-a expression had worsened OS and DFS rates than gastric cancer patients with low OPN-a expression, although the statistical analysis did not show significance among these groups. OPN-b showed a similar trend to OPN-a. In contrast, a higher OPN-c mRNA level was significantly associated with a better prognosis in patients with gastric cancer (Figure 1).

### 3.2. Association of OPN Splice variants’ Expression with Clinicopathological Features in Gastric Cancer

As shown in Table 2, the mRNA expression levels of the OPN splice variants were significantly higher in gastric cancer tissues than in normal tissues. No significant relationship was found between OPN splice variants’ expression and gender. Both the expression of OPN-a and OPN-c were found to increase with differentiation and in tumours with poor clinical outcomes, although these did not reach statistical significance. In addition, increased OPN-a levels were found to be significantly associated with tumour stage, when comparing N0 to N1 + 2 + 3, M0 to M1, and TNM1 + 2 to TNM3 + 4, respectively. However, the expression levels of OPN-c decreased with increasing lymph node status and this reached statistical significance when comparing N0 with N1 + 2 + 3.

The multivariate analysis unveiled that OPN-c was significantly correlated with both OS (*p* = 0.014) and RFS (*p* = 0.013) (Table 3 and Table 4). Likewise, OPN-b was significantly correlated with both OS (*p* = 0.026) and RFS (*p* = 0.046) indicated by multivariate analysis. However, OPN-a did not show a correlation with either OS or RFS by multivariate analysis. The univariate analysis showed similar trends.

### 3.3. Effects of OPN Splice Variants on In Vitro Functions of Gastric Cancer Cell Line HGC-27, Respectively

**Figure 2:**
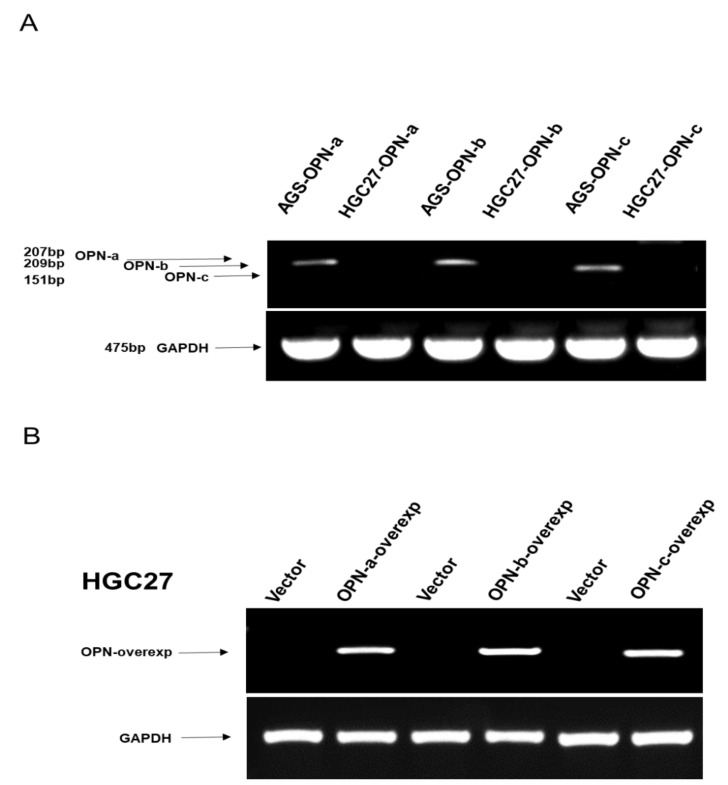
The expression of OPN genes in tissue from gastric cancer patients. (**A**) The expression of OPN-a, OPN-b, and OPN-c (207bp, 209bp and 151bp respectively) were detected via RT-PCR. As shown in Figure 2A, OPN isoforms a, b, and c were co-overexpressed in AGS cells and minimally expressed in HGC-27 cells. (**B**) The expression levels of OPN isoforms a, b, and c were considerably increased in OPN construct transfected cells, compared with vector control cells in HGC-27 cells (Figure 2B).

To investigate the expression pattern of OPN isoforms in gastric cancer, we firstly analysed their mRNA levels in gastric cell lines by RT-PCR. We found that OPN splice variants were upregulated in AGS cells, but barely detected in HGC-27 cells (Figure 2A). The expression levels of OPN isoforms were considerably increased in OPN isoforms construct transfected cells, compared with vector control cells in HGC-27 (Figure 2B). To further clarify the contribution of each OPN splice variant in gastric carcinogenesis, a series of in vitro cell function assays were performed.

### 3.4. Effect of OPN Splice Variants on the Proliferation of HGC-27 Cells

**Figure 3:**
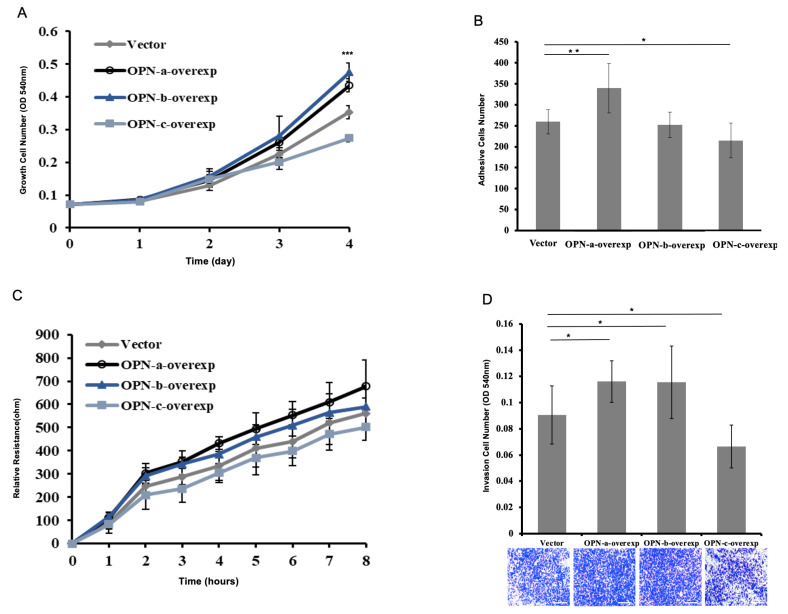
Effect of OPN variants on malignant properties of gastric cancer cells. (**A**) Compared with OPN-vector, OPN-a and OPN-b overexpression in HGC-27 increased in vitro growth, especially OPN-b, while OPN-c showed an inhibitory effect on tumor growth. (**B**) OPN-a overexpression in HGC-27 increased in vitro cellular adhesion, while OPN-c overexpression resulted in a remarkable reduction in the adhesion ability of HGC-27 cells. OPN-b did not show a noticeable effect on cellular adhesion. (**C**) Overexpression of OPN-a in HGC-27 cells showed an increased tendency in post-wound migration as indicated by the ECIS system. OPN-c overexpression resulted in a reduction in the migratory ability of HGC-27 cells. OPN-b did not show a noticeable effect on cellular migration. There were no statistically significant differences in both OPN-a-overexpression and OPN-c-overexpression groups at the 8th hour. (**D**) Compared with vector control cells, overexpression of OPN-a and OPN-b significantly promoted the transwell invasion. However, the high level of OPN-c remarkably decreased in vitro invasiveness (scale bars = 100 μm). * *p* < 0.05, ** *p* < 0.01, *** *p* < 0.001.

In the cell proliferation assay, cells with upregulated individual OPN-a and OPN-b both enhanced cell proliferation as compared with the corresponding vector control, especially OPN-b. Compared with vector control cells, overexpression of OPN-c resulted in an inhibition of cell growth (Figure 3A).

### 3.5. Effect of OPN Splice Variants on the Adhesion of HGC-27 Cells

As shown in Figure 3B, OPN-a overexpression in HGC-27 increased in vitro cellular adhesion, while OPN-c overexpression resulted in a remarkable reduction in the adhesion ability of HGC-27 cells.

### 3.6. Effect of OPN Splice Variants on the Migration of HGC-27 Cells

Overexpression of OPN-a in HGC-27 cells showed an increase in post-wound migration as indicated by the ECIS system; however, this did not reach statistical significance. OPN-c overexpression resulted in a slight reduction in the migratory ability of HGC-27 cells (Figure 3C).

### 3.7. Effect of OPN Splice Variants on the Invasion of HGC-27 Cells

Overexpression of OPN-a in HGC-27 cells showed a remarkable increase in invasion ability. A similar situation was also observed in OPN-b overexpressed cells, while overexpression of OPN-c could significantly decrease the number of migrated cells (Figure 3D).

### 3.8. Effect of OPN Splice Variants on ROS Generation of HGC-27 Cells Following Treatment with 5-Fu and Cisplatin, Respectively

**Figure 4:**
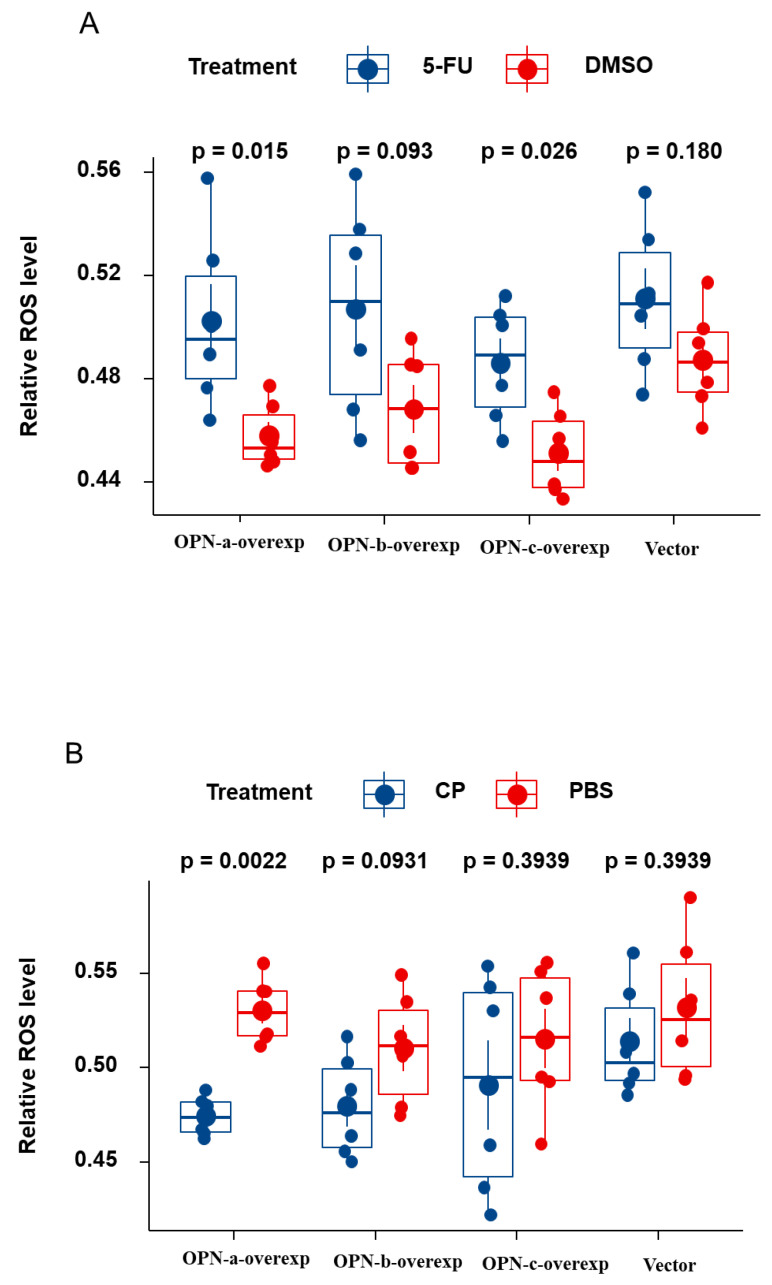
Effect of the OPN variants on the Cellular Reactive Oxygen Species (ROS) produced by gastric cells in response to chemotherapeutic drugs. (**A**) 5-Fu and its DMSO control. (**B**) Cisplatin (CP) and its PBS control.

To confirm that OPN splice variants in HGC-27 cells, following treatment with 5-Fu- and cisplatin, induced apoptosis mediated by ROS generation, we used the ROS detection assay kit to examine ROS production. Treatment with 5-Fu caused an increase in the ROS level in HGC-27 cells. Compared with OPN-vector, both OPN-a and OPN-c promoted ROS production after treatment with 5-Fu. However, cisplatin showed a diverse effect on ROS generation comparing to 5-Fu, and only OPN-a significantly decreased the level of ROS. Cisplatin and 5-Fu did not show an effect on ROS level in the OPN-b group and its vector-only control (Figure 4). 

### 3.9. Effect of OPN Splice Variants on Protein Structural Features

**Figure 5:**
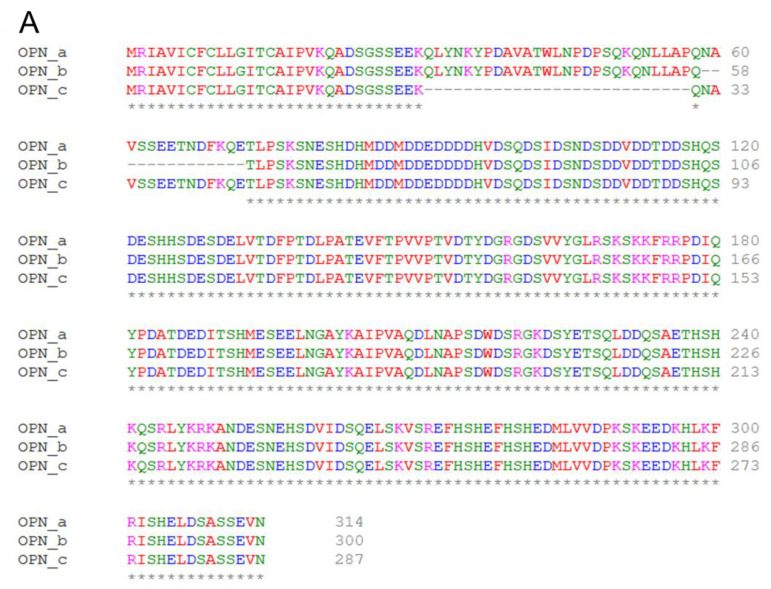

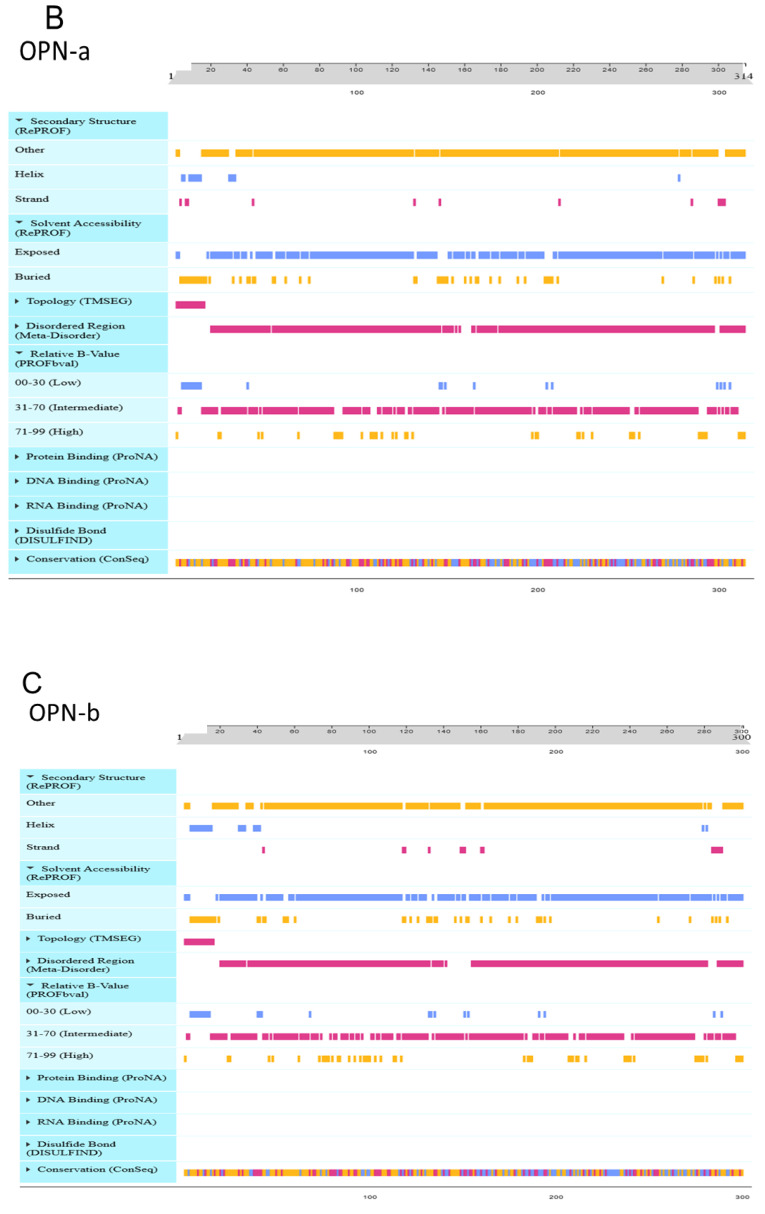

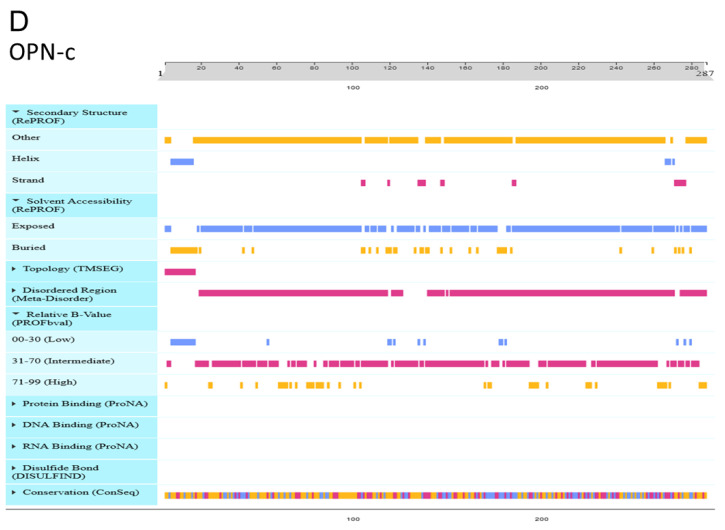
Prediction of the protein features of the OPN splice variants. (**A**) Protein sequences. (**B**) Protein features of OPN-a. (**C**) Protein features of OPN-b. (**D**) Protein features of OPN-c.

To investigate how the splice variants affect the protein structure, we first aligned the three variants. As shown in Figure 5A, the mismatch mainly occurred in the N-side of the protein sequences. OPN-b and OPN-c have different missing fragments compared to OPN-c which has the full sequence. The protein structure prediction showed that in the secondary structure, OPN-a forms 4 helixes and 8 strands (Figure 5B), OPN-b forms 5 helixes and 6 strands (Figure 5C), while OPN-c forms 3 helixes and 6 strands at different sites (Figure 5D).

## 4. Discussion

OPN is one of the known proteins that have been considered to correlate with tumourigenesis and progression of cancers, including gastric cancer [14,15,16,17,18]. Many recent studies show that OPN splice variant expression in malignancy is tissue-type specific and may have functional heterogeneity [19,20,21]. However, very few specific reports have elaborated the relationship between the expression of OPN isoforms and gastric cancer. In order to characterize the three OPN isoforms in gastric cancer, we firstly focused on the prognostic value of OPN splice variants in gastric cancer patients. Our results demonstrate that high OPN-a or OPN-b expression correlates with poor prognosis, which is opposite to OPN-c. Moreover, the increased expression of OPN-a, but not of OPN-b and OPN-c, is associated with adverse clinicopathological features. Together with these findings in gastric cancer tissues, we postulate that the abnormal alternative splicing, which results in elevated OPN-a expression, may intensively occur during the progression of GC, while OPN-c may prevent adverse clinical outcomes. A previous study also shows that OPN-a is highly expressed in gastric cancer, which is in line with our findings [22]. However, that study also suggests that OPN-c is highly expressed in gastric cancer, which is in contrast with our result. The inconsistency of the findings may be due to sampling sources, measurement sensitivity, or data normalization, which require further investigation using a large-scale cohort and more robust measurement and data analysis approach.

We next clarified the diverse malignant phenotypes of three OPN splice variants through in vitro cell function assays. We found that over-expression of OPN-a was linked to the elevation of proliferation, adhesion, invasion, and migration of HGC-27 cells in vitro. In contrast, following genetic overexpression of OPN-c, the properties of cellular proliferation, adhesion, invasion, and migration in HGC-27 cells were reduced. The results indicate that OPN splice variants may have functional heterogeneity in HGC-27 cells. As the heterogeneity of OPN splice variants has previously been described in other cancer types [23,24,25], our study further extends the observation of this phenomenon into gastric cancer. 

It has been demonstrated that OPN can promote cell survival by negatively regulating apoptosis in response to stress conditions, including exposure to anticancer agents; OPN splice variants might play distinct roles in this regard. A previous study suggests that acquired up-regulation of OPN-b and c isoforms might prevent conventional chemotherapy drug (Daunorubicin, Cytarabine and Idarubicin)-induced apoptosis in acute myeloid leukemia (AML) cells by upregulating the expression of AKT, VEGF, STAT3, CXCR4, and IL-6 [26]. In PC3 prostate cancer (PCa) cells, a study showed that OPN-c or OPN-b overexpression mediates resistance and cell survival features in response to docetaxel-induced cell death [27]. However, little evidence indicates the connection between OPN splice variants and chemotherapy drug-induced apoptosis in gastric cancer cells. Reactive oxygen species (ROS) production is considered a critical stressor that causes cell death, especially through the induction of apoptosis [28,29]. Herein, we investigated the association between the expression of three main OPN isoforms and chemotherapeutic drug-induced apoptosis in the HGC-27 cell line, which was evaluated by detecting ROS generation. Our study indicates that both OPN-a and OPN-c can effectively promote ROS production which is induced by 5-Fu when compared with corresponding controls, especially OPN-a. Meanwhile, in HGC-27 cells treated with cisplatin, overexpressed OPN-a significantly inhibits ROS production and OPN-c does not show an effect on ROS level.

It is worth noting that the changed trends of ROS in the two groups of drugs are the opposite. In the 5-Fu group, 5-Fu increased the level of ROS produced by OPN-vector cells, indicating that 5-Fu may promote apoptosis in HGC-27 cells by increasing the generation of ROS to a higher toxicity level, thus playing an anti-tumor effect. After the treatment with 5-Fu, in the HGC-27 cells with the over-expression of OPN-a and OPN-c, ROS production was significantly enhanced. This suggests that OPN-a and OPN-c variants play a role in promoting the anti-tumor effect of 5-Fu on HGC-27 cells by increasing the level of ROS. In contrast, in the cisplatin groups, cisplatin did not affect the levels of ROS produced by OPN-vector and OPN-c vector containing cells. However, cisplatin reduces the ROS level in HGC-27 cells overexpressing OPN-a. This implies that ROS may play a tumor-promoting role in cisplatin-treated HGC-27 cells, and overexpression of OPN-a may enhance the anti-tumor effect of cisplatin in HGC-27 cells by inhibiting the generation of ROS. Recent studies suggest that ROS seems to be a double-edged sword in the mediation of cancer [30,31,32]. On the one hand, ROS generation may promote tumor initiation and progression by activating oncogenes or inactivating tumor suppressor genes, regulating VEGF signal transduction pathways to promote angiogenesis, and activating AKT and PKC to induce cell invasion or metastasis [33,34]. However, on the other hand, ROS can increase the sensitivity of cancer cells to various death-inducing signalling pathways (such as apoptosis, autophagy, necrosis, etc.), thereby inhibiting tumor progression [35]. Therefore, the biological role of ROS in cancer is complicated. At present, our preliminary experimental results show that the expression of OPN-a and OPN-c can enhance the anti-tumor effect of 5-Fu on HGC-27 cells, while the expression of OPN-a can enhance the anti-tumor effect of cisplatin on HGC-27 cells. In the process, they may affect the balance between cellular ROS production and clearance. In the future, we will further elucidate possible molecular mechanisms by expanding cell lines, setting drug concentration gradients, and detecting signalling transduction. 

Differential effects of the three splice variants may be due to the distinctive functions of the spliced exons that individual OPN isoforms contain. For example, exon 4 encodes two glutamine residues essential for transglutaminase crosslinking. As exon 4 is absent in OPN-c, but not in OPN-a and OPN-b, OPN-c has no specific functions exerted by exon 4. In addition, deletion of exon 4 may alter the pattern of post-translational modifications (PTMs), resulting in functional modifications [36]. Protein prediction also suggests that the three OPN splice variants form different secondary structures. That might explain why these isoforms of OPN have cell-type-specific patterns. 

Although the present study, by using a large gastric cancer cohort, has shown the importance and the differential role of the three OPN variants in this rather lethal cancer, the study has its limits. First, the study was unable to carry out a protein-based investigation, due largely to the lack of suitable antibodies that can clearly distinguish the three variants. Fortunately, the minor differences of the variants can be distinguished by gene amplification studies such as the one carried out here. It is expected that antibodies of suitable quality will be developed in the near future to allow such studies to be carried out. Secondly, the study was unable to provide experimental information from a suitable in vivo tumour model or models. Information from in vivo models would provide an important link between the in vitro and clinical findings. It would be vital in the future to unveil the crystal structures of the OPN protein isoforms. This would allow in depth mechanistic investigation into the reasons behind the distinctive roles of the OPN variants in cancer and, importantly provide a suitable platform in search for druggable compounds. Furthermore, as OPN is considered an inflammatory sensing factor, it would be interesting to explore how OPN isoforms are involved in inflammation-mediated tumorigenesis in the stomach. With the development of antibodies specific to individual OPN isoforms, it may be worth examining whether the levels of the secretory OPN isoforms in patient plasma could be used to predict the progression of gastric cancer [37]. Although it is a challenge, appropriate in vivo gastric cancer models would be a prerequisite before we attempt to pinpoint the actual mechanisms of differential functions of OPN isoforms in vivo. Collectively, the study has provided some vital findings for the highly interesting variants of OPN and demonstrated that some of the variants are important clinical, prognostic indicators in gastric cancer. The study has laid the foundation for future work to be carried out, including searching for in-depth mechanisms of distinctive actions and for potential therapeutic targeting methods. 

## 5. Conclusions

In conclusion, we believe that this is the first work that shows that OPN splice variants have distinctive clinical and biological roles in gastric cancer. Exploring the implications of OPN splice-derived protein isoforms in gastric cancer could open up a novel avenue for the development of diagnostic and therapeutic approaches in patients with tumor malignancies.

## Figures and Tables

**Table 1:** Sequences for primers.

RT-QPCR
Primers for OPN-a(NM:001040058.2; Length:105 bp)
5′-ACAACAAATACCCAGATGCT-3′
5′-ACTGAACCTGACCGTACACATTGGTTTCTTCAGAGGAC-3′
Primers for OPN-b(NM:000582.3; Length:95 bp)
5′-ACAACAAATACCCAGATGCT-3′
5′-ACTGAACCTGACCGTACAGGACTTACTTGGAAGGGTCT-3′
Primers for OPN-c(NM:001040060.2; Length:87 bp)
5′-AAGTTCTGAGGAAAAGCAGA-3′
5′-ACTGAACCTGACCGTACACTTTCGTTGGACTTACTTGG-3′
Primers for GAPDH(NM: 002046.7; Length:93 bp)
5′-CTGAGTACGTCGTGGAGTC-3′
5′-ACTGAACCTGACCGTACACAGAGATGATGACCCTTTTG-3′
RT-PCR
Primers for OPN-a(NM:001040058.2; Length:207 bp)
5′-ATCTCCTAGCCCCACAGAAT-3′
5′-CATCAGACTGGTGAGAATCATC-3′
Primers for OPN-b(NM: 000582.3; Length:209 bp)
5′-ATCTCCTAGCCCCACAGAC-3′
5′-AAAATCAGTGACCAGTTCATCAG-3′
Primers for OPN-c(NM: 001040060.2; Length:151 bp)
5′-TGAGGAAAAGCAGAATGCTG-3′
5′-GTCAATGGAGTCCTGGCTGT-3′
Primers for GAPDH(NM: 002046.7; Length:475 bp)
5′- GGCTGCTTTTAACTCTGGTA -3′
5′- GACTGTGGTCATGAGTCCTT -3′

**Table 2:** The expression of OPN splice variants in tissue from gastric cancer patients. Analysis was performed by Mann–Whitney U test and results were considered statistically significant if *p* < 0.05.

Characteristics		n	OPN-a Median (IQ Range)	*p*-Value	OPN-b Median (IQ Range)	*p*-Value	OPN-c Median (IQ Range)	*p*-Value
Sample type	Tumor	324	19110 (513, 681494)	<0.0001	22 (1, 303)	<0.0001	338 (9, 8715)	0.0002
	Normal	324	491 (129, 2346)		0 (0, 1)		28 (7, 223)	
Gender	Male	231	15781 (490, 509248)	0.4447	18 (1, 255)	0.7282	280 (6, 6031)	0.5666
	Female	93	52657 (550, 1549284)		40 (0, 505)		601 (12, 14579)	
Infiltration depth	T1 + T2	42	5502 (48, 399965)	0.1116	8 (0, 402)	0.2819	74 (7,5 713)	0.4678
	T3 + T4	274	23948 (603, 762375)		25 (1, 297)		358 (9, 10974)	
Nodal status	N0	71	2967 (59, 579536)	0.0346	8 (0, 274)	0.2856	552 (9, 14259)	0.0226
	N1 + 2 + 3	247	29723 (1032, 850931)		25 (1, 363)		336 (6, 9106)	
M-staging	M0	282	13518 (323, 546163)	0.0076	19 (1, 275)	0.097	287 (6, 8292)	0.139
	M1	41	214420 (9820, 4845742)		44 (3, 1402)		439 (33, 15523)	
TNM staging	TNM1 + 2	85	5502 (63, 546163)	0.0385	8 (1, 287)	0.3448	433 (8, 10485)	0.0551
	TNM3 + 4	230	30629 (983, 758385)		25 (1, 338)		336 (9, 7539)	
Differentiation	High	1						
	High-Medium	6	51281(9947, 547813)		14.84(1.13, 41.01)		1932 (79, 82560)	
	Medium	62	11039 (55, 360955)	0.4234	41(2, 1584)	0.258	687 (4, 14259)	0.7000
	Medium-Low	82	54626 (989, 1141029)	0.8892	44 (1, 493)	0.2533	540 (14, 13658)	0.6071
	Low	138	15546 (734, 381642)	0.7801	11 (0, 231)	0.751	120 (6, 2892)	0.4134
Clinical outcome	Alive	120	16689 (305, 550306)	0.5139	22 (1, 248)	0.9832	1004 (24, 20290)	0.375
	Died	177	23867 (601, 842154)		22 (0, 607)		113 (3, 5596)	
	Disease-Free	106	16139 (239, 519513)	0.4325	20 (1, 246)	0.4857	724 (26, 13547)	0.3237
	Metastasis	14	118027 (1201, 8879,44)		83 (2, 731)		5044 (2, 65,790)	

**Table 3:** Multivariate and univariate analysis of overall survival (OS). Exp(B), exponentiation of the B coefficient.

	Multivariate vs. OS		Univariate vs. OS	
	*p* Value	Exp(B)	*p* Value	Exp(B)
Sex	0.34	0.718	0.882	0.963
Diagnosis	0.088	2.06	0.38	1.269
TNM	<0.001	5.322	<0.001	5.294
Location	0.478	1.071	0.836	1.013
Histology	0.106	1.329	0.296	0.924
Differentiation	0.464	0.863	0.312	1.145
Invasion	0.611	1.143	<0.001	0.591
Embolism	0.296	1.424	<0.001	3.095
OPN-a	0.855	1.066	0.38	1.255
OPN-b	0.026	2.675	0.064	1.753
OPN-c	0.014	0.446	0.009	0.54

**Table 4:** Multivariate and univariate analysis of relapse-free survival (RFS). Exp(B), exponentiation of the B coefficient.

	Multivariate vs. RFS		Univariate vs. RFS	
	Sig.	Exp(B)	Sig.	Exp(B)
Sex	0.456	0.766	0.92	1.026
Diagnosis	0.146	1.892	0.111	1.59
TNM	<0.001	5.916	<0.001	4.908
Location	0.488	1.072	0.725	1.024
Histology	0.172	1.276	0.946	1.005
Differentiation	0.468	0.861	0.203	1.193
Invasion	0.422	1.242	0.001	0.607
Embolism	0.335	1.401	<0.001	3.147
OPN-a	0.685	1.158	0.173	1.43
OPN-b	0.046	2.503	0.09	1.698
OPN-c	0.013	0.428	0.008	0.522

## Data Availability

Data reported in this study can be obtained from the authors upon reasonable request.
